# Inhibition of HER Receptors Reveals Distinct Mechanisms of Compensatory Upregulation of Other HER Family Members: Basis for Acquired Resistance and for Combination Therapy

**DOI:** 10.3390/cells10020272

**Published:** 2021-01-29

**Authors:** Daniela Gutsch, Robert Jenke, Thomas Büch, Achim Aigner

**Affiliations:** 1 Rudolf-Boehm-Institute for Pharmacology and Toxicology, Clinical Pharmacology, Faculty of Medicine, University of Leipzig, D-04107 Leipzig, Germany; daniela.gutsch@gmx.de (D.G.); robert.jenke@medizin.uni-leipzig.de (R.J.); thomas.buech@medizin.uni-leipzig.de (T.B.); 2 University Cancer Center Leipzig (UCCL), University Hospital Leipzig, D-04103 Leipzig, Germany

**Keywords:** HER2, HER3, erbB receptors, RNAi, miR-143, SATB1

## Abstract

Overexpression of members of the HER/erbB transmembrane tyrosine kinase family like HER2/erbB2/neu is associated with various cancers. Some heterodimers, especially HER2/HER3 heterodimers, are particularly potent inducers of oncogenic signaling. Still, from a clinical viewpoint their inhibition has yielded only moderate success so far, despite promising data from cell cultures. This suggests acquired resistance upon inhibitor therapy as one putative issue, requiring further studies in cell culture also aiming at rational combination therapies. In this paper, we demonstrate in ovarian carcinoma cells that the RNAi-mediated single knockdown of HER2 or HER3 leads to the rapid counter-upregulation of the respective other HER family member, thus providing a rational basis for combinatorial inhibition. Concomitantly, combined knockdown of HER2/HER3 exerts stronger anti-tumor effects as compared to single inhibition. In a tumor cell line xenograft mouse model, therapeutic intervention with nanoscale complexes based on polyethylenimine (PEI) for siRNA delivery, again reveals HER3 upregulation upon HER2 single knockdown and a therapeutic benefit from combination therapy. On the mechanistic side, we demonstrate that HER2 knockdown or inhibition reduces miR-143 levels with subsequent de-repression of HER3 expression, and validates HER3 as a direct target of miR-143. HER3 knockdown or inhibition, in turn, increases HER2 expression through the upregulation of the transcriptional regulator SATB1. These counter-upregulation processes of HER family members are thus based on distinct molecular mechanisms and may provide the basis for the rational combination of inhibitors.

## 1. Introduction

The HER/erbB transmembrane tyrosine kinase family comprises four members, HER1–HER4 (erbB1–erbB4). Receptor signalling is based on ligand-mediated receptor dimerization and subsequent phosphorylation, leading to the activation of downstream signal transduction. This includes the MAPK and PI3K-Akt pathways that promote cell growth, survival, invasion and angiogenesis and thus play pivotal roles in tumor cells (see e.g., [1] for review). The constitutive activation of HER2 (erbB2, neu) based on overexpression and/or mutation has been observed in various cancers, including ovarian carcinoma, and has been linked to disease progression, resistance to treatment and poor prognosis [2,3,4]. Epithelial ovarian cancer is typically found in postmenopausal women and is the commonest cause of gynaecological cancer-associated death. Most women present with advanced disease, where the standard of care (surgery and platinum-based chemotherapy; curative for most patients with early stage disease) shows only limited efficacy. More specifically, most women with advanced disease will experience tumor recurrence and chemoresistance (see e.g., [5] for review). Targeted therapies may thus offer urgently needed novel treatment options.

Consequently, the inhibition of HER2 tyrosine kinase activity is explored therapeutically in many cancers by employing low molecular weight tyrosine kinase inhibitors or monoclonal antibodies that block receptors, ligand binding or receptor dimerization (for review, see e.g., [6,7,8]). However, significant clinical responses are observed only in certain patients and tumor types, and in many cases they eventually suffer from resistance and disease progression [9,10,11]. In contrast to HER2, overexpression or mutation of HER3 does not occur so frequently. Nevertheless, despite its lack of intrinsic kinase activity, HER3 has been analyzed as a potential tumor driver and explored as a therapeutic target ([8,12] for review). For example, previous data suggest that the neuregulin/HER3 autocrine loop drives the growth of ovarian tumors in vivo, and HER3 was identified as a potential therapeutic target in ovarian cancer [13]. Furthermore, HER3 inhibition reverses transformation in HER2-positive tumors and induces tumor apoptosis [14,15]. Thus, several HER3-targeted approaches are in clinical trials [16].

Mechanisms of inherent or acquired resistance of tumors to HER-inhibiting therapies include somatic HER receptor mutations, compensatory signalling through activation of downstream signalling effectors or through receptors outside of the HER family, or increased expression of HER ligands ([17,18], see [19] for review). Yet, resistance is still a major subject of investigation, which may also be explained by the compensatory upregulation of other HER family members [20,21]. This is particularly relevant since heterodimerization plays an important role in the HER receptor network (see [1,7] for review). HER2 is recruited as a preferred partner that can potently amplify signalling through increasing ligand binding affinity, receptor recycling and/or receptor stability [22,23,24], while lacking a ligand-binding domain [25]. In contrast, HER3, which binds several growth factors (neuregulins) leading to very robust PI3K recruitment, shows a defective kinase activity, and HER3 thus relies on a heterodimeric partner for signalling [26]. In fact, HER2/HER3 heterodimers show particularly potent mitogenic signals and are the most transforming of this receptor network [22,24,27], emphasizing the relevance of HER2 and HER3 in cancer.

Beyond low molecular weight inhibitors or inhibitory antibodies, gene knockdown strategies can be explored as specific means of oncogene inhibition. RNA interference (RNAi) has been identified as a particularly potent mechanism of gene silencing that relies on small interfering RNAs (siRNAs) for sequence-specific mRNA cleavage and degradation [28]. However, the therapeutic application of RNAi critically depends on strategies for the delivery of siRNAs in vivo (see e.g., [29] for review). We have previously established polymeric nanoparticles based on polyethylenimine as a powerful strategy for siRNA delivery after systemic injection ([30,31,32]; [33] for review). This system also allows the combination of siRNAs for the simultaneous targeting of multiple genes. 

The high potency of HER2-containing heterodimers and the emerging relevance of HER3 in solid tumors prompted us to analyse the effects of a double inhibition approach of HER family members through RNA-mediated dual knockdown. Interestingly, beyond the enhancement of anti-tumor effects upon the simultaneous inhibition of HER2 + HER3, we also found that this double knockdown counter-acts autocrine cellular adaptation processes that are based on the rapid counter-upregulation of the respective other HER family members after HER2 or HER3 knockdown, respectively. These effects, which rely on distinct molecular pathways, are also observed upon HER2 or HER3 inhibition through treatment with the inhibitors (trastuzumab, TX-1-85-1). As a mechanism of secondary resistance, it must be expected that therapeutic effects of the inhibition of a single HER receptor are limited by the counter-upregulation of other family members. Thus, our data establish a cellular and molecular basis for a combined inhibition/knockdown strategy to achieve more profound therapeutic effects and avoid rapid cellular adaptation processes.

## 2. Materials and Methods

### 2.1. siRNAs, Plasmids, Tissue Culture, and Animals

Chemically unmodified siRNAs were obtained from Thermo Scientific (Hamburg, Germany) or Dharmacon (Epsom, UK). A list of all siRNAs can be found in Table S1. The SATB1 expression vector was purchased from OriGene (Rockville, MD) and the empty pRC-CMV (Invitrogen/Life Technologies, Darmstadt, Germany) was used as negative control (mock transfection). SKOV3 ovarian carcinoma cells were purchased from the American type culture collection (ATCC), and the stable SKOV3-Luc reporter cell line (luciferase under the control of an NFkB promoter) has been described previously [34]. Cells were cultivated in a humidified incubator under standard conditions (37 °C, 5% CO_2_) in IMDM (PAA, Cölbe, Germany) supplemented with 10% fetal calf serum (FCS). Athymic nude mice (Hsd:Athymic Nude-Foxn1^nu^) (nu/nu) were obtained from Harlan Winkelmann (Borchen, Germany) and kept at 23 °C in a humidified atmosphere with food and water ad libidum. Animal studies were approved by the Regierungspräsidium Giessen, Germany.

### 2.2. mRNA Preparation, miRNA Preparation and Quantitative RT-PCR

For the preparation of total RNA, the Tri reagent (PEQLAB, Erlangen, Germany) was used according to the manufacturer’s protocol. Tissues were homogenized prior to RNA preparation in 200 μL Tri reagent. MiRNA was isolated using the miRVana Kit (Ambion) according to the manufacturer’s protocol. Reverse transcription (RT) was performed using the RevertAid H Minus First Strand cDNA Synthesis Kit from Fermentas (St. Leon-Rot, Germany) as described previously [35]. Quantitative PCR was performed in a LightCycler from Roche (Penzberg, Germany) using the QuantiTect SYBR Green PCR kit (Qiagen, Hilden, Germany) according to the manufacturer’s protocol with 4.5 μL cDNA (diluted 1:10), 1 μL primers (5 μM) and 5 μL SYBR Green master mix. A pre-incubation for 15 min at 95 °C was followed by 55 amplification cycles: 10 s at 95 °C, 10 s at 55 °C and 10 s at 72 °C. The melting curve for PCR product analysis was determined by rapid cooling down from 95 °C to 65 °C, and incubation at 65 °C for 15 s prior to heating to 95 °C. To normalize for equal mRNA/cDNA amounts, PCR reactions with target-specific and with actin-specific primer sets were always run in parallel for each sample, and target levels were determined by the formula 2^CP(target)/^2^CP(actin)^ with CP = cycle number at the crossing point (0.3).

### 2.3. In Vitro Transfections and Growth Assays

SKOV3 cells were seeded at 1 × 10^6^, 5 × 10^5^ and 1 × 10^5^ cells (6-well plate) for harvest after 72, 120 and 168 h after transfection, respectively, at 3 × 10^5^ cells (12-well plate) for harvest at 48 h post transfection (NFκB reporter assay) and at 5 × 10^3^ cells/well (96-well plate, proliferation assay). Twenty-four hours after seeding, transfections were performed by the addition of the specific or non-specific siRNA (8 pmol, 4 pmol or 0.4 pmol siRNA/well of a 6-well, 12-well or 96-well plate, respectively), complexed with Interferin (Polyplus, Illkirch, France) according to the manufacturer’s protocol. Plasmid DNA was transfected at 1.5 μg or 0.5 μg/well of a 6-well or a 48-well plate, respectively, using PEI F25-LMW as described [31,34]. For RNA quantitation, cells from 6-well-plates were harvested at 72–168 h after transfection. For inhibitor treatment, SKOV3 cells were seeded at 1.5 × 10^6^ cells/well in 6-well plates, 1 day before treatment start with 5 μM TX-1-85-1 or with Herceptin for 72 h.

Studies of anchorage-dependent proliferation were carried out essentially as described previously [31] in the presence of IMDM/10% FCS. At the time points indicated, the numbers of viable cells in four wells were determined using a colorimetric assay according to the manufacturer’s protocol (Cell Proliferation Reagent WST-1, Roche Molecular Biochemicals, Mannheim, Germany). Anchorage-independent proliferation was studied in soft agar assays essentially as described previously [31]. Cells were transfected in 6-well plates with Interferin-complexed specific or non-specific siRNAs as described above and 24 h after transfection trypsinized and counted. Twenty-thousand cells in 0.35% agar (Bacto Agar, Becton Dickinson, Franklin Lakes, NJ, USA) were layered on top of 1 mL of a solidified 0.6% agar layer in a 35-mm dish. Growth media with 2% FCS were included in both layers. After 2–3 weeks of incubation, colonies more than 50 μm in diameter were counted by at least two blinded investigators. 

### 2.4. Apoptosis Assay

A commercially available bioluminescent caspase-3/7 assay (Caspase-Glo^®^ 3/7 assay, Promega, Mannheim, Germany) was used. The determination of apoptosis was performed in the 96-well format as recommended by the supplier, and luminescence was measured after 1 h incubation at 37 °C in the dark using a Fluostar Optima reader (BMG Labtec, Jena, Germany). 

### 2.5. ELISA, FACS Analysis and Immunohistochemistry

Human HER3 concentrations were determined from tumor lysates using the DuoSet ELISA Development System from R&D Systems (Abingdon, UK). For the preparation of tumor lysates, samples were homogenized in 500 μL PBS using a Dounce homogenizer, and after centrifugation at 13,000 rpm, supernatants were transferred into a fresh tube. Samples were diluted in reagent diluent (1% BSA in PBS), and the ELISA was performed according to the manufacturer’s protocol (R&D Systems). Absorbance was measured in an ELISA reader at 450 nm with background subtraction at 595 nm. Recombinant human HER3 (R&D) in appropriate buffers served as the standard.

For FACS analysis, SKOV3 cells were transfected as described above. Cells were trypsinized using 0.5 mL trypsin/well, mixed with 5 mL IMDM/10%FCS and centrifuged at 1000 rpm for 10 min. For extracellular staining, cells were resuspended in PBS/0.5% BSA at 500,000 cells/mL and transferred to a 1.5 mL vial. Primary antibodies against HER2 (c-erbB-2 Ab-2 (Clone 9G6.10)) were obtained from Neomarkers (Fremont, CA, USA) and diluted 1:250 in PBS/0.5% BSA. The HER3 antibody was obtained from GeneTex (Irvine, CA, USA) and diluted 1:500 in the same buffer. Antibody incubation was performed at 4 °C on a rolling shaker for 1–2 h. Cells were washed three times in PBS/0.5% BSA prior to incubation with a TexasRed-labeled anti-mouse IgG (Vector Labs) or a DyLight 594-labeled anti-rabbit IgG (VectorLabs) secondary antibody at 4 °C on a rolling incubator for 30 min in the dark. Cells were washed as above and the cell pellet was carefully resuspended in 300 μL PBS/0.5% BSA. Cells were measured by FACS (FACSCalibur, Becton-Dickinson) within 1 h.

Immunostaining of paraffin sections was performed essentially as described previously [31,35]. Briefly, after deparaffinization with xylene and rehydration with graded alcohols, sections were incubated in 10 mM citrate buffer (pH 7.4) at 90 °C for 15 min and endogenous peroxidases were inactivated with 0.3% hydrogen peroxide at 4 °C for 30 min. After blocking with 10% normal horse serum in PBST/2% BSA for 1 h at room temperature, the slides were incubated with mouse monoclonal anti-HER2 antibody (Dako, Hamburg, Germany) or rabbit polyclonal anti-SATB1 antibody, diluted 1:400 in PBST, at RT overnight in a wet chamber. For detection, biotinylated horse anti-(mouse-IgG) or goat anti-(rabbit-IgG) antibody (Vector Laboratories, Burlingame, CA, USA, 1:500) was applied for 1 h, and immunoreactivity on the sections was visualized using a streptavidin-biotin-peroxidase complex (ABC kit, Vector Laboratories) according to the manufacturer’s instructions, prior to treatment with the peroxidase substrate diaminobenzidine for obtaining a brown staining. For the assessment of protein levels, the percentage and intensity of brown staining across the whole tumor section was assessed by two independent, blinded investigators. 

### 2.6. Therapeutic Knockdown in Subcutaneous Tumor Xenografts 

PEI F25-LMW/siRNA complexes were prepared essentially as described previously [31,35]. Briefly, 100 pmol (1.3 μg) siRNA was dissolved in 80 μL 1 M HEPES/150 mM NaCl, pH 7.4, and incubated for 10 min. Twenty-two microliters of PEI F25-LMW (0.6 μg/μL) [31] was dissolved in 80 μL of the same buffer, and after 10 min pipetted to the siRNA solution. After 30 min incubation, the mixture was aliquoted and stored frozen. Prior to use, complexes were thawed and incubated for 1 h at room temperature.

2.5 × 10^6^ SKOV3 cells in 150 μL PBS were injected s.c. into both flanks of athymic nude mice. When solid tumors were established, mice were randomized into treatment groups (8 mice per group). Treatment was performed by i.p. injection of 0.77 nmoles (10 μg) PEI F25-LMW-complexed specific or PEI-complexed nonspecific siRNA at the time points specified in the figure (see arrows). Tumor volumes were monitored every 2–3 days at the time points shown in the figure and, upon termination of the experiment 1 d after the last treatment, mice were sacrificed and tumors removed. Pieces of each s.c. tumor were immediately fixed in 10% paraformaldehyde for paraffin embedding or snap-frozen for RNA preparation, miRNA preparation or ELISA (see above).

## 3. Results

### 3.1. The Single Knockdown of HER2 or HER3 Leads to the Rapid Counter-Upregulation of Other HER Family Members

**Figure 1:**
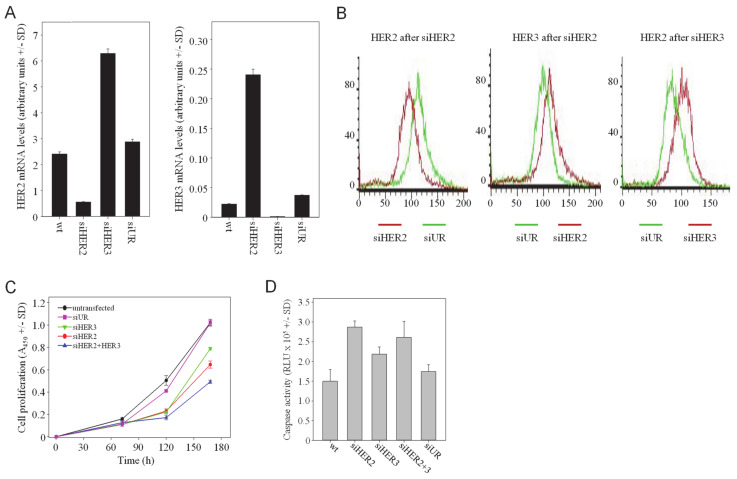
HER single knockdown leads to the counter-upregulation of the respective other HER family member, and combined HER2/HER3 knockdown leads to enhanced antitumor effects. (**A**) SKOV3 cells were transfected with siRNAs as indicated in the figures and analyzed for mRNA levels of HER2 (left) and HER3 (right). (**B**) Alterations of HER2 and HER3 protein levels, as determined by flow cytometry. (**C**) Anti-proliferative and (**D**) pro-apoptotic effects upon siRNA-mediated single or double knockdown of HER2 and/or HER3.

For HER2 or HER3 inhibition, transient siRNA-mediated gene knockdown by RNAi was chosen. The selected siRNAs showed knockdown efficacies 80–90% for targeting HER2 (siHER2) or siHER3 in SKOV3 cells (Figure 1A). Strikingly, the siRNA-mediated reduction of HER3 led to a marked and rapid >2-fold upregulation of HER2 mRNA (siHER3 in Figure 1A, left). Vice versa, HER2 knockdown resulted in an even more profound ~7-fold increase in HER3 mRNA levels (siHER2 in Figure 1A, right). These effects on HER mRNAs also translated into parallel alterations in HER receptor protein levels, as determined by FACS analysis. Specific knockdown of HER2 resulted in the upregulation of HER3, while siHER3 transfection led to increased HER2 protein levels (Figure 1B). Similar findings were obtained in SKBr-3 breast carcinoma cells, with >3-fold higher HER2 mRNA levels upon siHER3 transfection and a comparable HER3 mRNA upregulation as a consequence of HER2 knockdown (Figure S1A). Taken together, this demonstrates the interplay between the expression of HER2 and HER3 and suggests counter-upregulation processes upon a given single knockdown. Notably, changes in HER receptor levels are not due to receptor internalization or recycling since these effects were observed already on the mRNA level.

### 3.2. Combined RNAi-Mediated Knockdown of HER Family Members

The rapid upregulation of an oncogenic RTK, i.e., HER2 or HER3, may counteract tumor cell inhibitory effects of HER3 or HER2 inhibition/knockdown, respectively. This also emphasizes the potential benefit of combined HER2 and HER3 knockdown, which was analyzed next. The combination of two siRNAs for double knockdown revealed differences regarding the efficacy of target gene downregulation as compared to the single treatment. The combined siHER2 + siHER3 transfection led to residual HER2 levels similar to siHER2 single knockdown (~26%; Figure S2A), indicating that the siHER3-mediated upregulation of HER2 could be fully compensated in the double knockdown setting and did not affect HER2 knockdown efficacy. This, however, was not seen in the case of HER3 upregulation. Here, HER3 knockdown efficacies were lower upon HER2 + HER3 double knockdown when compared to siHER3 alone (60% vs. 20%; Figure S2B). Note that these effects were not based on different siRNA concentrations, non-specific siRNA effects or RISC overload, since in the case of every single knockdown non-specific siRNAs were added in order to transfect with identical amounts of total siRNA. 

### 3.3. Additive Effects of Double Knockdown of HER Family Members on Cell Proliferation and Apoptosis

To explore whether the simultaneous targeting of more than one HER family member leads to increased anti-tumor effects, assays for anchorage-dependent proliferation were performed. While the transfection of SKOV3 cells with siHER3 led to a moderate ~20% reduction of cell proliferation over 7 d as compared to non-transfected (wt) or negative control siRNA-transfected (siUR) cells, the single knockdown of HER2 was slightly more efficient with 40% reduced cell proliferation (Figure 1C). Double treatment of cells with siHER2 + siHER3 resulted in increased effects with >50% reduced proliferation in SKOV3 cells. A more efficient inhibition was obtained in SKBr-3 cells, which shows higher endogenous HER3 expression (Figure S1B). HER3 single knockdown was less efficient than siHER2 transfection (~50% vs. ~75% inhibition, respectively). Notably, however, siHER2 + siHER3 double transfection essentially abrogated cell proliferation, emphasizing the biological relevance of HER2/HER3 heterodimers. This indicates the impact of the parallel knockdown of HER2 and HER3 despite comparably low HER3 expression levels in SKOV3 wt cells [36] and losses in HER3 knockdown efficacy upon double transfection (see Figure S2B). 

These findings were confirmed in soft-agar assays, monitoring anchorage-independent growth and thus mimicking more closely the in vivo situation. While HER3 single knockdown was again the least efficient and siHER2 resulted in a ~70% reduction of colony formation, the maximum inhibitory effect on colony growth (~80%) was observed upon HER2 + HER3 double knockdown (Figure S2C). 

Profound effects were also observed when measuring apoptosis. Beyond cell growth and motility, HER receptors have been shown to affect cell survival as well. To analyse the effects of a HER (double) knockdown on apoptosis, caspase-3/7 activation was determined in SKOV3 cells. The single HER2 or HER3 knockdown led to a ~2-fold or 1.5-fold increase in apoptosis, respectively. Caspase-3/7 activity was not further enhanced upon siHER2 + siHER3 double transfection, identifying HER3 inhibition as a major driver of apoptosis induction (Figure 1D). 

Still, we conclude that HER2 + HER3 double knockdown leads to more profound tumor cell inhibition over single knockdowns, thus confirming the HER2/HER3 heterodimer as particularly relevant. 

### 3.4. Enhanced Anti-Tumor Effects In Vivo upon Therapeutic HER2/HER3 Double Knockdown through Systemic Treatment of Mice with siRNAs

**Figure 2:**
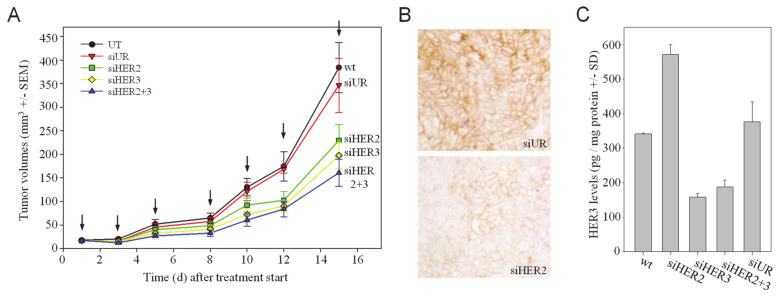
Systemic treatment of mice bearing s.c. SKOV3 tumor xenografts with PEI/siRNA complexes for HER knockdown. (**A**) Anti-tumor effects of HER single or double knockdown by systemic (i.p.) injection of PEI/siRNA complexes. (**B**) Immunohistochemical analysis of HER2 protein levels in tumors from different treatment groups. Representative examples of stained slides without counterstain are shown. (**C**) Analysis of tumor lysates for HER3 protein levels.

To test whether the HER2 + HER3 double knockdown also leads to more profound antitumor effects in a therapeutic setting in vivo, mice bearing s.c. tumor xenografts were treated with an RNAi therapy protocol. More specifically, siRNAs were complexed with polyethylenimine (PEI) for the formation of polymeric nanoparticles mediating siRNA protection, cellular uptake and intracellular release as described previously [31,37]. Upon establishment of tumors from s.c.-injected SKOV3 wildtype cells, mice were treated three times per week by intraperitoneal injection of 10 μg PEI-complexed specific (siHER2, siHER3) or non-specific (siUR) siRNAs, or left untreated (UT). The monitoring of tumor sizes revealed a ~30–40% reduction of tumor growth upon single knockdown of HER2 or HER3, respectively (Figure 2A). The combined treatment with siHER2 + siHER3 exerted a more profound effect with a ~50% reduction of tumor growth. In this experimental setting, it was not possible to determine a survival advantage after specific treatment since the study was terminated for all mice at the same time point. 

To confirm that these anti-tumor effects were based on the specific knockdown of the target genes, HER2 levels were analyzed by immunohistochemistry and revealed reduced HER2 protein levels upon PEI/siHER2 treatment to the negative control PEI/siUR group (Figure 2B). Likewise, the determination of HER3 protein levels in tumor lysates by ELISA revealed the specific inhibition of HER3 expression in the siHER3 treatment groups, with a ~60% knockdown efficacy (Figure 2C). Notably, upon HER2 single knockdown a significant increase in HER3 protein (~1.6-fold) was observed (Figure 2C). Thus, this confirmed the previous in vitro findings regarding the (counter-) upregulation processes upon HER knockdown also in the in vivo situation and prompted us to further investigate the underlying molecular mechanisms.

### 3.5. The microRNA miR-143 Mediates the Counter-Upregulation of HER3 upon HER2 Knockdown

MicroRNAs have been recognized as important regulators of gene expression, acting on their target mRNA through translational repression or mRNA degradation. Simultaneously, miRNAs are often differentially expressed e.g., in normal vs. pathological tissues including tumors, which allows their use as biomarkers and has been shown to be of functional relevance. Previously, the expression of several miRNAs has been associated with HER2 receptor status, including miR-143, which is significantly upregulated in HER2-negative vs. HER2-positive tumors [38] and is considered as tumor-suppressing miRNA frequently downregulated in tumors (see e.g., [35,39]). In silico analysis by target scan (www.targetscan.org) identified HER3 as one target for miR-143, prompting us to analyse the possible role of miR-143 in the HER2/HER3 interplay in more detail.

**Figure 3:**
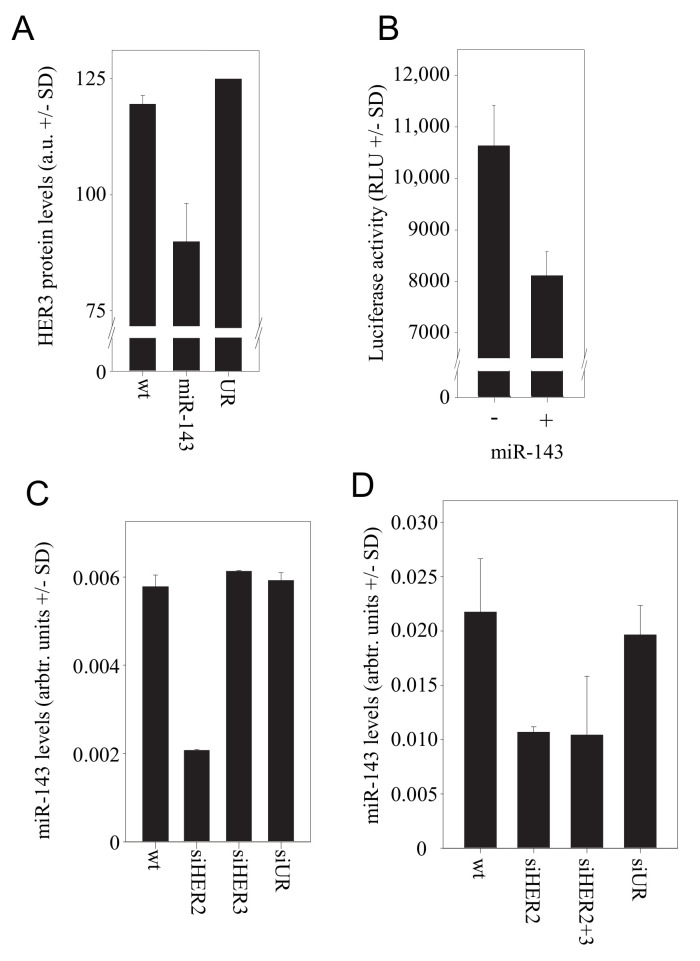
Role of miR143 in the HER2/HER3 regulation. (**A**) Effect of miR-143 transfection on HER3 protein levels. (**B**) HER3 is a direct target of miR-143 as determined in a luciferase reporter assay. (**C**) Upon siRNA-mediated HER2 knockdown, a marked reduction of miR-143 levels is observed. (**D**) MiR-143 levels in the tumor xenografts from the PEI/siRNA therapy experiment.

Indeed, transfection of the cells with miR-143 resulted in a ~30% downregulation of HER3 on the protein level (Figure 3A).

To confirm HER3 as a target of miR-143, we generated a construct comprising the 3′-UTR of HER3 fused to a luciferase reporter gene. Luciferase expression of the reporter construct in SKOV3 cells was reduced by ~20% upon transfection of miR-143 (Figure 3B). Vice versa, the siRNA-mediated HER2 knockdown in SKOV3 cells led to a very profound >60% reduction in the level of miR-143 (Figure 3C), suggesting that the expression of this miRNA is regulated by HER2-mediated signalling. Results were again confirmed in samples of the in vivo therapy study. The analysis of tumor xenografts for miR-143 levels revealed indeed a >40% downregulation of miR-143 upon treatment with PEI/siHER2 or upon PEI/siRNA-mediated double knockdown of HER2 and HER3 (Figure 3D). Taken together, this demonstrates the downregulation of miR-143 upon HER2 knockdown, leading in turn to the de-repression of HER3 expression, and thus establishes miR-143 as a link between HER2 and HER3 levels.

### 3.6. The Transcriptional Regulator SATB1 Is Responsible for the Inverse Correlation between HER3 and HER2

**Figure 4:**
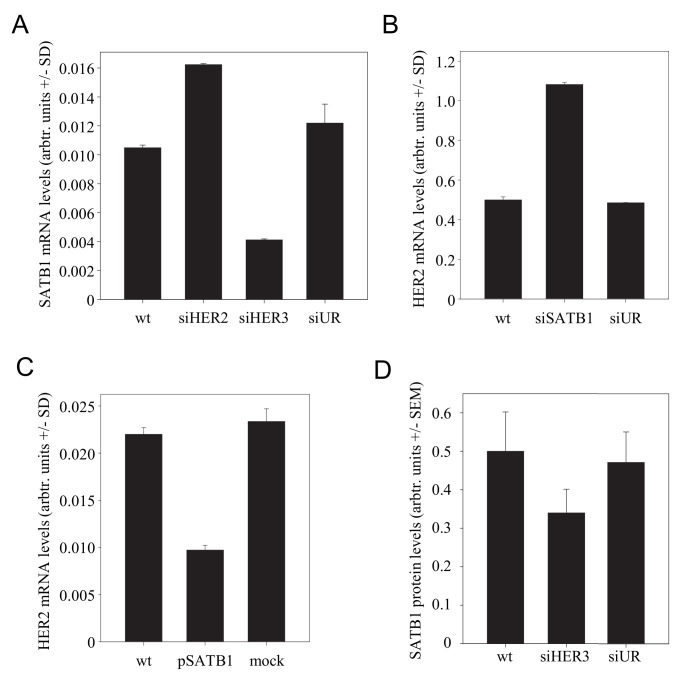
Effect of SATB1 in the HER3/HER2 regulation network. (**A**) Alterations in SATB1 mRNA levels upon knockdown of HER2 or HER3. (**B**) SiRNA-mediated SATB1 knockdown leads to increased HER2 mRNA levels. (**C**) SATB1 overexpression decreases HER2 mRNA levels. (**D**) SATB1 levels in the tumor xenografts from the PEI/siRNA therapy experiment, as determined by immunohistochemistry.

The chromatin organiser and transcriptional regulator “Special AT-rich binding protein 1” (SATB1) has been shown to be expressed during tumorigenesis and to affect the gene expression profile of breast cancer cells [40]. It supports an aggressive phenotype and promotes tumor growth and metastasis in various tumor entities (see [41] for review). Previously, functional profiling had revealed that HER receptors can be regulated by SATB1, with results, however, being dependent on the cell line [40]. We thus hypothesized that SATB1 may be involved in the mutual regulation of HER receptor expression. Notably, when we analyzed SKOV3 cells after HER3 knockdown, a marked >60% downregulation of SATB1 was observed (Figure 4A). This SATB1 inhibition led in turn to the induction of HER2 expression, as demonstrated by a >2-fold increase in HER2 mRNA levels upon siRNA-mediated SATB1 knockdown (Figure 4B). We confirmed this finding in the reverse experiment by SATB1 overexpression, which led to reduced HER2 levels (Figure 4C). No SATB1 effect was observed on HER3 expression. Taken together, this establishes a connection specifically between HER3 and HER2.

The regulation of SATB1 was also detected in vivo. Since a previous study had demonstrated SATB1 expression by stroma [42], we employed immunohistochemical staining for the determination of SATB1 expression in the tumor tissues rather than the determination of SATB1 levels from tumor lysates. Indeed, in PEI/siHER3-treated mice, decreased SATB1 protein levels were found (Figure 4D).

Notably, the in vitro transfection experiments also demonstrated that HER2 knockdown led to increased SATB1 levels (Figure 4A). This supports the previous finding that HER2 negatively affects HER3 expression (note the increased HER3 expression upon siHER2 transfection in Figure 1A) and explains this SATB1 upregulation. In summary, this completes the system of mutual counter-regulation mechanisms based on miR-143 and SATB1 (see below).

### 3.7. The Counter-Regulatory Network of HER Family Members Is also Observed upon Small Molecule Inhibitor- or Antibody-Mediated Inhibition

**Figure 5:**
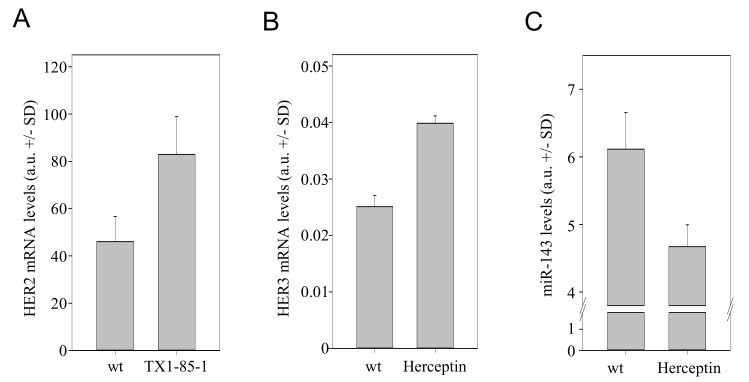
Effects of TX-1-85-1 or trastuzumab (Herceptin) on the HER regulation network. SKOV3 cells were analyzed for (**A**) HER2 levels upon treatment with the inhibitor of HER3 phosphorylation, TX-1-85-1, or treated with Herceptin and analyzed for (**B**) HER3 and (**C**) miR-143 levels.

We next tested whether our findings from in vitro and in vivo knockdown experiments are also observed when using clinically established HER inhibitors. Indeed, when applying the inhibitor of HER3 phosphorylation, TX1-85-1, in SKOV3 cells, an ~1.8-fold increase in HER2 mRNA levels was observed (Figure 5A). This also indicates that the inhibition of HER signalling, with receptor protein levels remaining unchanged, is sufficient for counter-upregulation effects. For HER2 inhibition, trastuzumab (anti-HER2; Herceptin) has been approved for cancer therapy. In line with the above knockdown results, the trastuzumab-mediated inhibition of HER2 led to a >1.5-fold upregulation of HER3 (Figure 5B) as well as to reduced miR-143 levels (Figure 5C). Taken together, this confirms our above data from knockdown experiments and emphasizes their potential clinical relevance since the observed effects are not limited to RNAi-based knockdown approaches.

### 3.8. Model of the Network of Mutual and Compensatory Regulation of HER Expression

**Figure 6:**
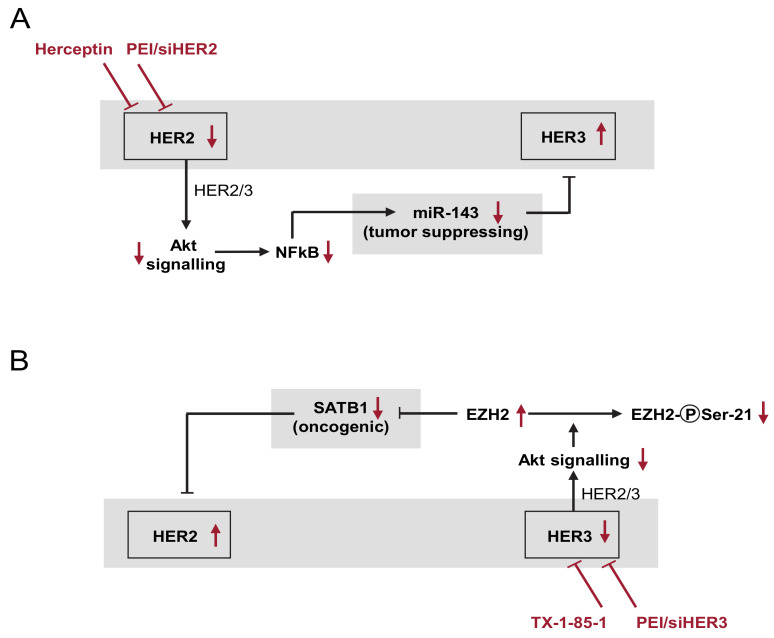
Schematic overview of the network of HER counter-(up)regulation processes (details: see text). (**A**) Effects upon the PEI/siHER2-mediated knockdown or the trastuzumab (Herceptin)-mediated inhibition of HER2, leading to HER3 upregulation. (**B**) HER3 knockdown or TX1-85-1-mediated inhibition with subsequent HER2 upregulation. Red arrows indicate the effects upon the respective inhibition or knockdown.

Based on our data from knockdown and inhibitor experiments in vitro and in vivo, we propose a network of HER counter-regulation processes, which is depicted in Figure 6A,B. HER2 knockdown (siHER2; Figure 6A) leads to reduced Akt signalling as indicated by reduced Akt phosphorylation (Figure S3A,B). Akt in turn has been shown to upregulate the transcriptional activity of NFκB (nuclear factor-κB) by inducing its phosphorylation and the subsequent degradation of IκB (inhibitor of κB) [43]. This was confirmed in our studies in an SKOV3 reporter cell line containing a luciferase expression plasmid under the control of an NFκB reporter. Indeed, a profound >50% reduction of NFκB-driven luciferase expression was observed upon siRNA-mediated HER2 knockdown, thus indicating reduced NFκB activity (Figure S3C). Since miR-143 expression is driven by NFkB as well [44], the decreased NFkB activity leads to the downregulation of the tumor-suppressing miR-143. Decreased miR-143 levels, in turn, result in translational de-repression and thus in HER3 upregulation (see Figure 3).

On the other hand, Figure 6B shows that HER3 induces the expression of the oncogenic transcription factor SATB1 via Akt signalling and EZH2. SATB1 negatively affects HER2 levels (see Figure 4).

In agreement with this model based on siRNA-mediated knockdown, trastuzumab (Herceptin) treatment leads, through miR-143 downregulation, to higher HER3 expression (see Figure 5B,C), and the HER3 inhibitor mimics siHER3 effects by upregulating HER2 (see Figure 5A).

## 4. Discussion

When employing HER2-directed antibodies or tyrosine kinase inhibitors in cancer treatment, either in combination with chemotherapy or as single agents, therapeutic resistance typically occurs within months of starting therapy (see [19] for review). Several mechanisms of resistance have been described (see Introduction) which, unfortunately, limit the therapeutic success in the clinics.

In this paper, we describe a counter-(up)regulation network between HER2 and HER3, acting beyond already known resistance mechanisms. For our analyses, we employed RNAi-mediated knockdown of individual family members, allowing the precise dissection of any given member’s contribution to these processes. However, it should be noted that we observed similar effects upon inhibition of HER2 or HER3 with trastuzumab or the low molecular weight inhibitor TX-1-85-1, respectively, supporting the clinical relevance of our findings. Still, one limitation of our study may be that our results primarily rest on one cell line, although key findings were also reproduced in another cell line derived from breast cancer (see Figure S1). Our data on HER3 upregulation are in line with previous results in trastuzumab-resistant HER2-overexpressing BT-474 breast carcinoma cells generated in vivo that showed higher levels of phosphorylated HER3 [45]. Notably, however, we demonstrate that these mechanisms act at early time points after antibody- or RNAi-mediated HER2 inhibition, indicating distinct intracellular processes rather than the selection of a subset of tumor cells that eventually (re-)grow. This is in line with, and explains, the observation that HER3 mRNA and protein levels are upregulated already after short-term inhibition of HER2 with the tyrosine kinase inhibitors gefitinib or lapatinib [20,46], and that, after a transient inhibition, HER3 signalling resumes and persists despite continued exposure to drugs like gefinitib, erlotinib or AG825 [46].

Notably, the underlying mechanisms of interdependent regulation of HER expression described in this study rely on molecules that have already been identified as oncologically relevant. MiR-143 is down-regulated in various tumors, suggesting its (inhibitory) involvement in tumorigenesis [47]. In this study, we validate HER3 as a direct target of miR-143, as previously suggested by the identification of specific targeting sites for miR143 in the 3′-UTR of the HER3 gene [48]. This is in line with, and may help explain, the tumor-suppressing role of this miRNA. As shown previously [20,46], changes in steady-state HER3 signalling upon gefitinib or lapatinib treatment occur upon loss of Akt signal transduction, and indeed, miR-143 expression is driven by NFkB [44] that is activated through Akt. Thus, beyond the FoxO2A-dependent HER3 upregulation [20] or miR-125a/b sequestration by the HER2 3′-UTR leading to HER2 mRNA de-repression [21], miR-143 and its direct action on HER3 described here provide another link between HER2 and HER3 expression (see Figure 6A). This may well explain the previously observed ‘signal buffering’ [46] and suggests that HER2 and HER3 inhibitors should be used in combination (see below).

On the other hand, SATB1 is overexpressed during tumorigenesis and alters the gene expression profile of breast cancer cells, supporting an aggressive phenotype promoting tumor growth and metastasis ([40]; [41] for review). In line with this, SATB1 has been described to upregulate HER3 [40]. While this finding is supported by our data, our model provides evidence for an indirect mechanism of SATB1-mediated HER3 upregulation, based on the reduction of HER2 expression followed by miR-143 downregulation. Of note, our data are in contrast to previous findings demonstrating the SATB1-mediated upregulation of HER2 [40]. This, however, was observed in the cell lines MDA-MB-231 and HS-578T, both of which are models of HER2-negative tumors, while we employed HER2-positive cell lines, SKBr-3 and SKOV3. Thus, again, results may be dependent on the cellular and molecular context. We employed HER2-positive cell lines since the HER2 inhibition is obviously most relevant here, and notably, the effects described in this paper were observed in both cell lines. On the other hand, the at first glance counter-intuitive finding that the proto-oncogene SATB1 mediates the downregulation of the oncogenic HER2 can be explained by the counter-upregulation of HER3 upon HER2 inhibition. Again, this indicates the pivotal role of HER3 as particularly potent member of the HER family. In line with this, the HER3/neuregulin autocrine loop has been described as an attractive target in ovarian cancer [13], and the role of HER3 as a potential tumor driver and therapeutic target has attracted increasing interest ([8,12,16] for review). Finally, our results also demonstrate that HER3 upregulates SATB1 (see Figure 4). Again, this can be explained through the action of Akt, which has been shown to phosphorylate the ‘Enhancer of Zeste homolog 2′ (EZH2), thus suppressing its methyltransferase activity [49]. This in turn may release the epigenetic repression of SATB1 by EZH2 [50].

Our data, as well as previous findings on compensatory mechanisms that enhance HER2/HER3 signalling [20,46], also emphasize the usefulness of double inhibition approaches. While some antibodies already address this issue by inhibiting heterodimer formation rather than individual receptors or homodimers, it should be noted that trastuzumab has been shown to exert preferential activity in breast tumors driven by HER2 homodimers [51], while it is unable to block ligand-induced formation of HER1/HER2 or HER2/HER3 heterodimerization [52,53]. Consequently, synergistic effects have been observed upon the combination of trastuzumab with lapatinib or with pertuzumab [54,55,56,57].

Aiming at combination therapies, alternative approaches of HER inhibition may provide advantages over antibodies or low molecular weight inhibitors. RNAi has been recognized as promising since it allows the highly specific knockdown of any given target gene, when using appropriate siRNAs, and their defined combination in one formulation. The major bottleneck so far, however, has been the efficient non-viral delivery of therapeutic siRNAs in vivo. Several strategies have been developed that may provide an avenue towards the therapeutic exploration of siRNAs (see e.g., [29] for review). Our PEI-based delivery platform combines several important features, i.e., (i) the protection of the siRNA molecule against degradation, (ii) the cellular uptake in its nanoparticle formulation, (iii) its intracellular endosomal/lysosomal escape probably due to the ‘proton-sponge effect’, and (iv) its subsequent release from the non-covalent PEI/siRNA complex into the cytoplasm ([33] for review). Thus, polyethylenimines or modified PEIs may represent a promising platform for therapeutic siRNA delivery. We have shown previously that the PEI/siRNA complexes as employed here are highly biocompatible and demonstrated the absence of toxicity (nephro-/hepatotoxicity, stimulation of the innate immune system, other effects on mouse well-being or behaviour) [31]. Notably, most recently we have also extended our PEI/siRNA nanoparticle studies towards patient-derived xenograft (PDX) models and demonstrated efficacy as well (Karimov et al., under review). Of note, this nanoparticle approach also allows to freely combine different siRNAs aiming at the simultaneous inhibition of more than one target gene which may indeed lead to improved antitumor efficacy, also based on counteracting mechanisms of secondary resistance as described here. This, however, will rely on the identification of optimal (siRNA) combinations for rational tumor therapy. While cell lines are unlikely to recapitulate the entire molecular complexity of tumors and HER signal transduction, these and other studies demonstrate their usefulness for identifying critical pathways and mechanisms of acquired resistance.

## Data Availability

The data presented in this study are available in this article and its associated Supplementary Materials (see above).

## Supplementary Materials

The following are available online at https://www.mdpi.com/2073-4409/10/2/272/s1, Figure S1: (A) HER2 (left) and HER3 mRNA levels (right) upon transfection of SKBr3 cells with siRNAs as indicated in the figures. (B) Anti-proliferative effects upon siRNA-mediated single or double knockdown of HER2 and/or HER3 in SKBr3 cells. Figure S2: Knockdown efficacies upon transfection of SKOV3 cells with single or combined siRNAs. (A) HER2 and (B) HER3 mRNA levels. Equal amounts of specific and of total siRNAs were used for transfection. (C) Inhibition of anchorage-independent proliferation of SKOV3 cells in soft agar assays upon siRNA-mediated single or combined HER2/3 knockdown. Figure S3: Effects of siHER2 or siHER3 transfection on phospho-Akt levels and NFκB activity. (A) Quantitation of flow cytometry results, (B) original data from siHER2 transfection, (C) NFκB-driven luciferase expression in a stable SKOV3-NFκB-Luciferase reporter cell line. Table S1: SiRNA sequences.

## References

[1] Yarden Y., Sliwkowski M.X. (2001). Untangling the ErbB signalling network. Nature reviews. Mol. Cell Biol..

[2] Hynes N.E., MacDonald G. (2009). ErbB receptors and signaling pathways in cancer. Curr. Opin. Cell Biol..

[3] Slamon D.J., Clark G.M., Wong S.G., Levin W., Ullrich A., McGuire R.L. (1987). Human breast cancer: Correlation of relapse and survival with amplification of the HER2/neu oncogene. Science.

[4] Ross J.S., Fletcher J.A. (1998). The HER-2/neu oncogene in breast cancer: Prognostic factor, predictive factor, and target for therapy. Stem Cells.

[5] Jayson G.C., Kohn E.C., Kitchener H.C., Ledermann J.A. (2014). Ovarian cancer. Lancet.

[6] Lurje G., Lenz H.J. (2009). EGFR signaling and drug discovery. Oncology.

[7] Citri A., Yarden Y. (2006). EGF-ERBB signalling: Towards the systems level. Nature reviews. Mol. Cell Biol..

[8] Lyu H., Han A., Polsdofer E., Liu S., Liu B. (2018). Understanding the biology of HER3 receptor as a therapeutic target in human cancer. Acta Pharm. Sin. B.

[9] Vogel C.L., Cobleigh M.A., Tripathy D., Gutheil J.C., Harris L.N., Fehrenbacher L., Slamon D.J., Murphy M., Novotny W.F., Burchmore M. (2002). Efficacy and safety of trastuzumab as a single agent in first-line treatment of HER2-overexpressing metastatic breast cancer. J. Clin. Oncol..

[10] Kaufman B., Trudeau M., Awada A., Blackwell K., Bachelot T., Salazar V., DeSilvio M., Westlund R., Zaks T., Spector N. (2009). Lapatinib monotherapy in patients with HER2-overexpressing relapsed or refractory inflammatory breast cancer: Final results and survival of the expanded HER2+ cohort in EGF103009, a phase II study. Lancet Oncol..

[11] Baselga J., Albanell J., Ruiz A., Lluch A., Gascon P., Guillem V., Gonzalez S., Sauleda S., Marimon I., Tabernero J.M. (2005). Phase II and tumor pharmacodynamic study of gefitinib in patients with advanced breast cancer. J. Clin. Oncol..

[12] Kiavue N., Cabel L., Melaabi S., Bataillon G., Callens C., Lerebours F., Pierga J.Y., Bidard F.C. (2020). ERBB3 mutations in cancer: Biological aspects, prevalence and therapeutics. Oncogene.

[13] Sheng Q., Liu X., Fleming E., Yuan K., Piao H., Chen J., Moustafa Z., Thomas R.K., Greulich H., Schinzel A. (2010). An activated ErbB3/NRG1 autocrine loop supports in vivo proliferation in ovarian cancer cells. Cancer Cell.

[14] Holbro T., Beerli R.R., Maurer F., Koziczak M., Barbas C.F., Hynes N.E. (2003). The ErbB2/ErbB3 heterodimer functions as an oncogenic unit: ErbB2 requires ErbB3 to drive breast tumor cell proliferation. Proc. Natl. Acad. Sci. USA.

[15] Lee-Hoeflich S.T., Crocker L., Yao E., Pham T., Munroe X., Hoeflich K.P., Sliwkowski M.X., Stern H.M. (2008). A central role for HER3 in HER2-amplified breast cancer: Implications for targeted therapy. Cancer Res..

[16] Jacob W., James I., Hasmann M., Weisser M. (2018). Clinical development of HER3-targeting monoclonal antibodies: Perils and progress. Cancer Treat Rev..

[17] Mishra R., Hanker A.B., Garrett J.T. (2017). Genomic alterations of ERBB receptors in cancer: Clinical implications. Oncotarget.

[18] Sampera A., Sanchez-Martin F.J., Arpi O., Visa L., Iglesias M., Menendez S., Gaye E., Dalmases A., Clave S., Gelabert-Baldrich M. (2019). HER-Family Ligands Promote Acquired Resistance to Trastuzumab in Gastric Cancer. Mol. Cancer Ther..

[19] Garrett J.T., Arteaga C.L. (2011). Resistance to HER2-directed antibodies and tyrosine kinase inhibitors: Mechanisms and clinical implications. Cancer Biol. Ther..

[20] Garrett J.T., Olivares M.G., Rinehart C., Granja-Ingram N.D., Sanchez V., Chakrabarty A., Dave B., Cook R.S., Pao W., McKinely E. (2011). Transcriptional and posttranslational up-regulation of HER3 (ErbB3) compensates for inhibition of the HER2 tyrosine kinase. Proc. Natl. Acad. Sci. USA.

[21] Li X., Xu Y., Ding Y., Li C., Zhao H., Wang J., Meng S. (2018). Posttranscriptional upregulation of HER3 by HER2 mRNA induces trastuzumab resistance in breast cancer. Mol. Cancer.

[22] Pinkas-Kramarski R., Soussan L., Waterman H., Levkowitz G., Alroy I., Klapper L., Lavi S., Seger R., Ratzkin B.J., Sela M. (1996). Diversification of Neu differentiation factor and epidermal growth factor signaling by combinatorial receptor interactions. EMBO J..

[23] Graus-Porta D., Beerli R.R., Daly J.M., Hynes N.E. (1997). ErbB-2, the preferred heterodimerization partner of all ErbB receptors, is a mediator of lateral signaling. EMBO J..

[24] Wallasch C., Weiss F.U., Niederfellner G., Jallal B., Issing W., Ullrich A. (1995). Heregulin-dependent regulation of HER2/neu oncogenic signaling by heterodimerization with HER3. EMBO J..

[25] Klapper L.N., Glathe S., Vaisman N., Hynes N.E., Andrews G.C., Sela M., Yarden Y. (1999). The ErbB-2/HER2 oncoprotein of human carcinomas may function solely as a shared coreceptor for multiple stroma-derived growth factors. Proc. Natl. Acad. Sci. USA.

[26] Guy P.M., Platko J.V., Cantley L.C., Cerione R.A., Carraway K.L. (1994). Insect cell-expressed p180erbB3 possesses an impaired tyrosine kinase activity. Proc. Natl. Acad. Sci. USA.

[27] Alimandi M., Romano A., Curia M.C., Muraro R., Fedi P., Aaronson S.A., Di Fiore P.P., Kraus M.H. (1995). Cooperative signaling of erbB3 and erbB2 in neoplastic transformation and human malignancies. Oncogene.

[28] Zamore P.D., Tuschl T., Sharp P.A., Bartel D.P. (2000). RNAi: Double-stranded RNA directs the ATP-dependent cleavage of mRNA at 21 to 23 nucleotide intervals. Cell.

[29] Kim B., Park J.H., Sailor M.J. (2019). Rekindling RNAi Therapy: Materials Design Requirements for In Vivo siRNA Delivery. Adv. Mater..

[30] Hampl V., Martin C., Aigner A., Hoebel S., Singer S., Frank N., Sarikas A., Ebert O., Prywes R., Gudermann T. (2013). Depletion of the transcriptional coactivators Megakaryoblastic Leukemia 1 and 2 abolishes hepatocellular carcinoma xenograft growth by inducing oncogene-induced senescence. EMBO Mol. Med..

[31] Hobel S., Koburger I., John M., Czubayko F., Hadwiger P., Vornlocher H.P., Aigner A. (2010). Polyethylenimine/small interfering RNA-mediated knockdown of vascular endothelial growth factor in vivo exerts anti-tumor effects synergistically with Bevacizumab. J. Gene Med..

[32] Hendruschk S., Wiedemuth R., Aigner A., Topfer K., Cartellieri M., Martin D., Kirsch M., Ikonomidou C., Schackert G., Temme A. (2011). RNA interference targeting survivin exerts antitumoral effects in vitro and in established glioma xenografts in vivo. Neuro Oncol..

[33] Hobel S., Aigner A. (2013). Polyethylenimines for siRNA and miRNA delivery in vivo. Wiley interdisciplinary reviews. Nanomed. Nanobiotechnol..

[34] Urban-Klein B., Werth S., Abuharbeid S., Czubayko F., Aigner A. (2005). RNAi-mediated gene-targeting through systemic application of polyethylenimine (PEI)-complexed siRNA in vivo. Gene Ther..

[35] Ibrahim A.F., Weirauch U., Thomas M., Grunweller A., Hartmann R.K., Aigner A. (2011). MicroRNA replacement therapy for miR-145 and miR-33a is efficacious in a model of colon carcinoma. Cancer Res..

[36] Moasser M.M., Basso A., Averbuch S.D., Rosen N. (2001). The tyrosine kinase inhibitor ZD1839 (“Iressa”) inhibits HER2-driven signaling and suppresses the growth of HER2-overexpressing tumor cells. Cancer Res..

[37] Aigner A. (2007). Nonviral in vivo delivery of therapeutic small interfering RNAs. Curr. Opin. Mol. Ther..

[38] Mattie M.D., Benz C.C., Bowers J., Sensinger K., Wong L., Scott G.K., Fedele V., Ginzinger D., Getts R., Haqq C. (2006). Optimized high-throughput microRNA expression profiling provides novel biomarker assessment of clinical prostate and breast cancer biopsies. Mol. Cancer.

[39] Motoyama K., Inoue H., Takatsuno Y., Tanaka F., Mimori K., Uetake H., Sugihara K., Mori M. (2009). Over- and under-expressed microRNAs in human colorectal cancer. Int. J. Oncol..

[40] Han H.J., Russo J., Kohwi Y., Kohwi-Shigematsu T. (2008). SATB1 reprogrammes gene expression to promote breast tumour growth and metastasis. Nature.

[41] Fromberg A., Engeland K., Aigner A. (2018). The Special AT-rich Sequence Binding Protein 1 (SATB1) and its role in solid tumors. Cancer Lett..

[42] Iorns E., Hnatyszyn H.J., Seo P., Clarke J., Ward T., Lippman M. (2010). The role of SATB1 in breast cancer pathogenesis. J. Natl. Cancer Inst..

[43] Bai D., Ueno L., Vogt P.K. (2009). Akt-mediated regulation of NFkappaB and the essentialness of NFkappaB for the oncogenicity of PI3K and Akt. Int. J. Cancer.

[44] Zhang X., Liu S., Hu T., Liu S., He Y., Sun S. (2009). Up-regulated microRNA-143 transcribed by nuclear factor kappa B enhances hepatocarcinoma metastasis by repressing fibronectin expression. Hepatology.

[45] Ritter C.A., Perez-Torres M., Rinehart C., Guix M., Dugger T., Engelman J.A., Arteaga C.L. (2007). Human breast cancer cells selected for resistance to trastuzumab in vivo overexpress epidermal growth factor receptor and ErbB ligands and remain dependent on the ErbB receptor network. Clin. Cancer Res..

[46] Sergina N.V., Rausch M., Wang D., Blair J., Hann B., Shokat K.M., Moasser M.M. (2007). Escape from HER-family tyrosine kinase inhibitor therapy by the kinase-inactive HER3. Nature.

[47] Wang Y., Lee C.G. (2009). MicroRNA and cancer--focus on apoptosis. J. Cell. Mol. Med..

[48] Yan X., Chen X., Liang H., Deng T., Chen W., Zhang S., Liu M., Gao X., Liu Y., Zhao C. (2014). miR-143 and miR-145 synergistically regulate ERBB3 to suppress cell proliferation and invasion in breast cancer. Mol. Cancer.

[49] Cha T.L., Zhou B.P., Xia W., Wu Y., Yang C.C., Chen C.T., Ping B., Otte A.P., Hung M.C. (2005). Akt-mediated phosphorylation of EZH2 suppresses methylation of lysine 27 in histone H3. Science.

[50] Lei L., Lu L., Xiang L., Xue-song W., De-pei L., Chih-chuan L. (2010). Epigenetic repression of SATB1 by polycomb group protein EZH2 in epithelial cells. Chin. Med. Sci. J..

[51] Ghosh R., Narasanna A., Wang S.E., Liu S., Chakrabarty A., Balko J.M., Gonzalez-Angulo A.M., Mills G.B., Penuel E., Winslow J. (2011). Trastuzumab has preferential activity against breast cancers driven by HER2 homodimers. Cancer Res..

[52] Cho H.S., Mason K., Ramyar K.X., Stanley A.M., Gabelli S.B., Denney D.W., Leahy D.J. (2003). Structure of the extracellular region of HER2 alone and in complex with the Herceptin Fab. Nature.

[53] Agus D.B., Akita R.W., Fox W.D., Lewis G.D., Higgins B., Pisacane P.I., Lofgren J.A., Tindell C., Evans D.P., Maiese K. (2002). Targeting ligand-activated ErbB2 signaling inhibits breast and prostate tumor growth. Cancer Cell.

[54] Xia W., Gerard C.M., Liu L., Baudson N.M., Ory T.L., Spector N.L. (2005). Combining lapatinib (GW572016), a small molecule inhibitor of ErbB1 and ErbB2 tyrosine kinases, with therapeutic anti-ErbB2 antibodies enhances apoptosis of ErbB2-overexpressing breast cancer cells. Oncogene.

[55] Nahta R., Hung M.C., Esteva F.J. (2004). The HER-2-targeting antibodies trastuzumab and pertuzumab synergistically inhibit the survival of breast cancer cells. Cancer Res..

[56] Baselga J., Cortes J., Im S.A., Clark E., Ross G., Kiermaier A., Swain S.M. (2014). Biomarker analyses in CLEOPATRA: A phase III, placebo-controlled study of pertuzumab in human epidermal growth factor receptor 2-positive, first-line metastatic breast cancer. J. Clin. Oncol..

[57] Scheuer W., Friess T., Burtscher H., Bossenmaier B., Endl J., Hasmann M. (2009). Strongly enhanced antitumor activity of trastuzumab and pertuzumab combination treatment on HER2-positive human xenograft tumor models. Cancer Res..

