# Supplementary material for: Inhibition of HER Receptors Reveals Distinct Mechanisms of Compensatory Upregulation of Other HER Family Members: Basis for Acquired Resistance and for Combination Therapy
Source: Cells. 2021 Jan 29;10(2):272. doi: 10.3390/cells10020272 (PMC7911202; doi:10.3390/cells10020272)
Supplement: Supplementary file 1 [file cells-10-00272-s001.pdf]

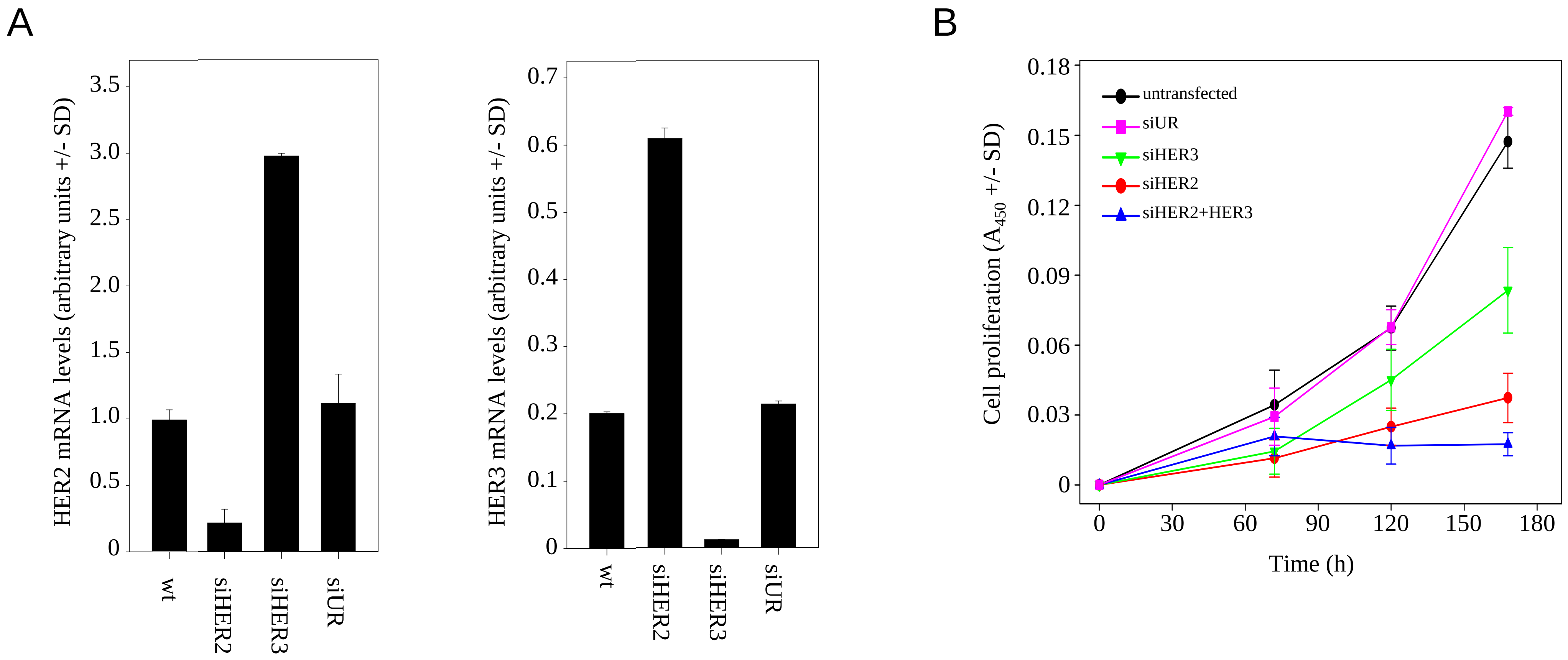

Gutsch et al., Fig. S1

A

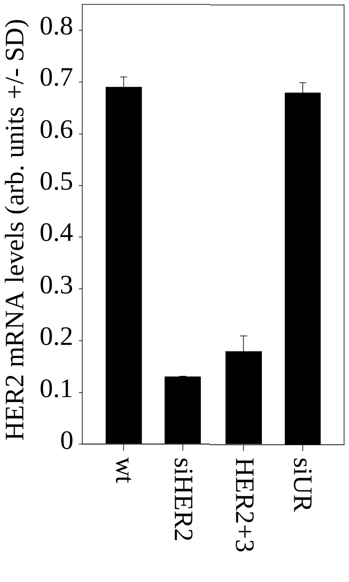

B

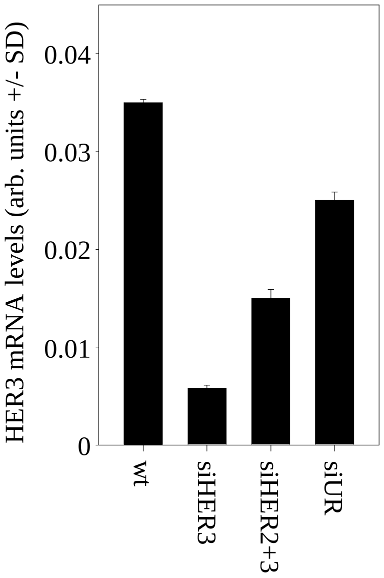

C

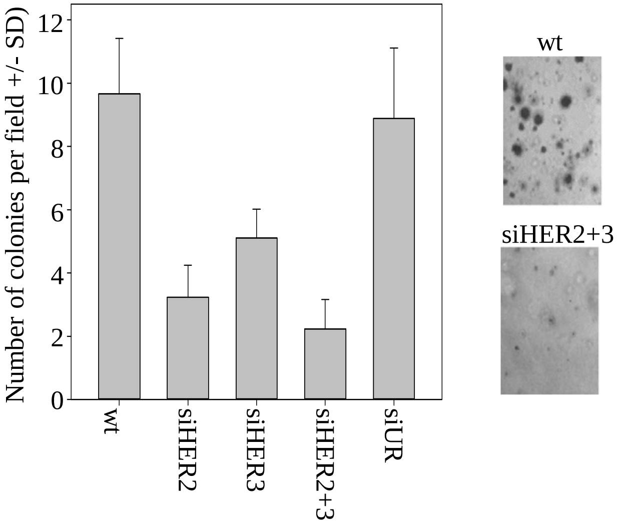

Gutsch et al., Fig. S2

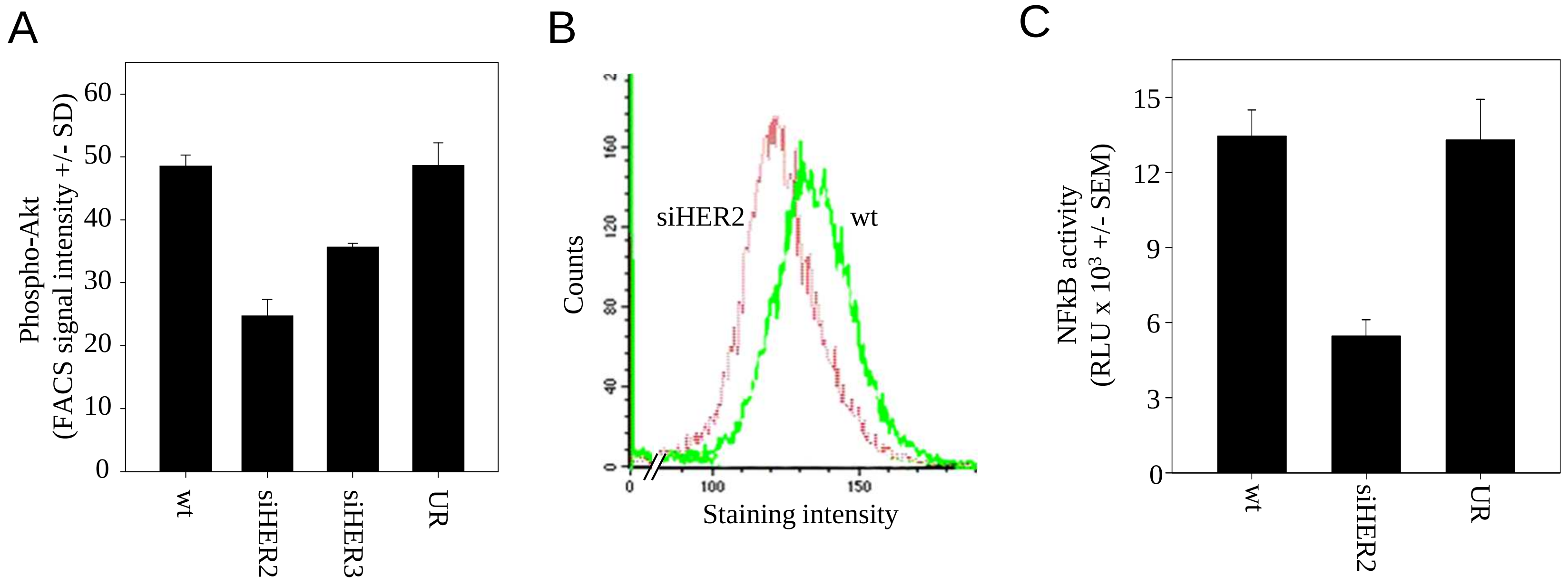

Gutsch et al., Fig. S3

Suppl. Table 1: SiRNA sequences

siRNA	Manufacturer	Sequence (1=sense; 2=antisense)
siHer1_55	Thermo Scientific, Hamburg	1:GGUGGUCCUUGGGAAUUUGdTdT<br>2:CAAAUUCCCAAGGACCACCdTdT
siHer2_08	Thermo Scientific, Hamburg	1:GCCUGAAZAZGZGAACCAGdTdT<br>2:CUGGUUCACAUAUUCAGGCdTdT
siHer3_02	Thermo Scientific, Hamburg	1:CCUUGAGAUUGUGCUCACGdTdT<br>2:CGUGAGCACAAUCUCAAGGdTdT
siSAT1	Sigma-Aldrich, Taufkirchen	1:GCUUCAAGAUGUGUAUCAUdTdT<br>2:AUGAUACACAUCUUGAAGCdTdT
siUR (siLuc3)	Thermo Scientific, Hamburg	1:CUUACGCUGAGUACUUCGAdTdT<br>2:UCGAAGUACUCAGCGUAAGdTdT
